# Pneumoproteins in Offshore Drill Floor Workers

**DOI:** 10.3390/ijerph16030300

**Published:** 2019-01-23

**Authors:** Niels E. Kirkhus, Bente Ulvestad, Lars Barregard, Øivind Skare, Raymond Olsen, Yngvar Thomassen, Dag G. Ellingsen

**Affiliations:** 1 National Institute of Occupational Health, 0033 Oslo, Norway; bente.ulvestad@stami.no (B.U.); oivind.skare@stami.no (Ø.S.); raymond.olsen@stami.no (R.O.); yngvar.thomassen@stami.no (Y.T.); dag.ellingsen@stami.no (D.G.E.); 2 Department of Occupational and Environmental Medicine, University of Gothenburg, SE-405 30 Gothenburg, Sweden; lars.barregard@amm.gu.se

**Keywords:** CC-16, SP-D, CRP, oil mist

## Abstract

The aim was to assess pneumoproteins and a certain biomarker of systemic inflammation in drill floor workers exposed to airborne contaminants generated during drilling offshore, taking into consideration serum biomarkers of smoking, such as nicotine (S-Nico) and cotinine. Blood samples of club cell protein 16 (CC-16), surfactant protein D (SP-D) and C-reactive protein (CRP) were collected before and after a 14-day work period from 65 drill floor workers and 65 referents. Air samples of oil mist, drilling mud components and elemental carbon were collected in person. The drill floor workers were exposed to a median air concentration of 0.18 mg/m^3^ of oil mist and 0.14 mg/m^3^ of airborne mud particles. There were no differences in the concentrations of CC-16 and SP-D across the 14-day work period and no difference between drill floor workers and referents at baseline after adjusting for differences in sampling time and smoking. CRP decreased across the work period. There was a strong association between the CC-16 concentrations and the time of sampling. Current smokers with S-Nico > detection limit (DL) had a statistically significantly lower CC-16 concentration, while smokers with S-Nico < DL had CC-16 concentrations similar to that of the non-smokers. Fourteen days of work offshore had no effect on serum pneumoprotein and CRP concentrations. However, the time of blood sampling was observed to have a strong effect on the measured concentrations of CC-16. The effect of current smoking on the CC-16 concentrations appears to be dependent on the S-Nico concentrations.

## 1. Introduction

Offshore drill floor workers are exposed to airborne contaminants composed of constituents of drilling fluids (mud). Mud is either a water- or oil-based complex chemical mixture [1] and exposure to mud has traditionally been surveilled by oil mist and oil vapor measurements [2,3,4,5,6,7]. There has been a substantial decline in exposure as assessed by oil mist air measurements in the Norwegian offshore sector from an average of 4.3 mg/m^3^ in 1989–1997 to 0.54 mg/m^3^ in 1998–2004 [8]. Furthermore, non-volatile mud components are present in their workroom atmosphere but these components have rarely been measured although mud containing aerosols may have alkaline properties [1,9,10]. Drill floor workers are engaged in manual work at the drill floor and in the mud handling area. At the drill floor, where the drilling engine is located, drill-pipes are added to the drill string and mud is pumped into the drill hole. The mud circulating between the drill hole and the drilling rig is used in multiple applications, such as for stabilization of the drill hole and for removal of the drill cuttings. The returning mud is separated from the cuttings in the mud handling area of the rig with use of shale shakers [10]. 

To our knowledge, only one study has addressed lung disorders in drill floor workers that are potentially caused by exposure to oil mist, other mud components or other airborne chemical exposures, such as strong cleaning fluids [11]. A slight reduction in pulmonary function across a 14-day work period was suggested in that study. Molecular pathological epidemiological studies can provide insight into the intermediate processes between the state of non-disease and serious disease by using various biomarkers and thus, contribute to the prevention of disease at an early stage [12]. No previous studies have examined pneumoproteins in drill floor workers. Altered levels of lung-specific proteins measured in serum have been proposed as markers for pulmonary injury [13]. However, oil mist exposed rats experienced a substantial reduction of SP-D in their bronchioalveolar lavage fluid [14]. It is believed that these proteins move passively across the epithelial barrier into the blood in higher amounts after epithelial injury [13]. Club cell protein (CC-16; previously termed Clara cell protein 16) and surfactant protein D (SP-D) are two pneumoproteins found in the serum that are commonly used as biomarkers for pulmonary injury.

The anti-inflammatory protein CC-16 is predominantly secreted from the nonciliated bronchiolar club cells but can also be found in lower amounts in non-pulmonary tissues [15,16]. In humans, SP-D is most highly expressed in the distant airways and alveoli, mainly in type 2 pneumocytes, but also in club, goblet and tracheo-bronchial glandular cells [17,18]. These proteins have mainly immune defense properties and are involved in the regulation of inflammation. SP-D promotes lysis of microbes, phagocytosis of pathogens and modulates cytokine and reactive oxygen networks and the function of various inflammatory cells, such as macrophages and lymphocytes [18]. Increased serum concentrations of SP-D have been observed in a number of human pulmonary diseases, including sarcoidosis, cystic fibrosis, asthma and chronic obstructive pulmonary disease [19,20]. Mice lacking SP-D spontaneously develop emphysema [21]. In vitro studies have indicated that CC-16 can attenuate inflammation by inhibiting phospholipase A2 activity. Inhibition of monocyte and polymorphonuclear chemotaxis and phagocytosis are also functions of CC-16 [15]. 

Several studies have suggested that the serum levels of acute phase reactant C-reactive protein (CRP) may be increased as a result of exposure to particulate matter, although a recent review concluded that the evidence is not conclusive [22]. It has been suggested that alveolar macrophages phagocytizing particulate matter express pro-inflammatory cytokines that interact with neighboring airway epithelial cells [23]. This may result in systemic inflammatory responses with induction of acute phase reactants, such as CRP, mediated through increased secretion of IL-6 [23].

The aim of this study was to investigate if drill floor workers exposed to oil mist and other chemical components when drilling for oil and gas in the Norwegian North Sea sector would experience alterations in serum concentrations of well-known biomarkers for pulmonary damage (SP-D and CC-16) and systemic inflammation (CRP). A further aim was to assess if smoking as assessed by serum concentrations of nicotine and cotinine would affect biomarker concentrations. This study is part of a larger study on occupational exposure and pulmonary health of offshore drill floor workers [10,11].

## 2. Material and Methods

### 2.1. Study Design and Participants

We designed a 14-day follow-up study of currently exposed drill floor workers and non-exposed referents working at offshore oil drilling rigs in the Norwegian North Sea sector. The study was restricted to employees working on rigs served by helicopter transport from Sola airport (Stavanger, Norway). Workers employed by seven different companies on a total of six moveable and four stationary drilling rigs and two stationary non-drilling rigs agreed to participate. Informed consent was collected from all participants before inclusion. The study was conducted in accordance with the Declaration of Helsinki and the study was approved by the South East Regional Ethical Committee for Medical Research, Norway (REK 2009/186a).

The participants were examined at the heliport of Sola airport before they were transported to the rigs and after they returned from the rigs after their 14-day work period. Participants for the study were successively recruited before the outbound flight until the target numbers of 65 drill floor workers and 65 referents were examined. Further details on inclusion criteria have been previously published [10]. Many participants live far away from Sola airport and may have to travel far to reach the heliport. Only two participants could be examined before each helicopter flight due to limited time between helicopter transport and travel to and from the participants’ homes. The drill floor workers were required to carry air sampling equipment during their offshore stay for the collection of occupational exposure data. The timing of the health examinations in this study depended on the departure schedules of the helicopters transporting workers to and from the rigs. For logistic and safety reasons, the helicopter brings a new crew offshore before the old crew is returned on-shore. Thus, it was anticipated that it could be difficult to examine the participants on the same time of the day when they were travelling off-shore and on-shore. The time of sampling was therefore considered as a covariate in the statistical analysis. 

The main work tasks of drill floor workers on the drill floor included manual procedures related to drill pipe connection and disconnection during drilling and cleaning up spills with pressure washers. They also operated the shale shakers, which included monitoring of mud flow and removal of cuttings, changing shaker screens regularly and cleaning the screens with pressure washers. They may also have visited the mud pit and the mud pump areas [1,10].

Offshore working and accommodation environments may vary from domestic environments onshore and therefore, a reference group of other offshore workers was additionally added. Referents were selected from work groups without any known regular exposure to occupationally generated air contaminants, including managers, administrative personnel, technicians, radio operators, medics, catering personnel (cooks excluded), deck crews, mechanics and production workers.

### 2.2. Examinations

Background data were collected by a self-administered validated questionnaire [24]. The questions included the occurrence of respiratory symptoms, the habits of smoking and tobacco snuffing and information on current and previous diseases. Ten mL of whole blood was collected for determination of pneumoproteins, CRP and biomarkers of smoking and snuffing from the cubital vein in vacutainers without additives (Becton Dickinson and Company, Franklin Lakes, NJ, USA). The samples were centrifuged at 2000 *g* for 15 min after resting for 45 min. The serum was then pipetted into two 4.0-mL NUNC^®^ polypropylene cryotubes (Thermo Fisher Scientific, Watham, MA, USA) and frozen immediately at −20 °C before long-term storage at −80 °C at the National Institute of Occupational Health (Oslo, Norway). Blood could not be collected at baseline for one referent.

### 2.3. Laboratory Analysis

Serum concentrations of CC-16 and SP-D were measured with commercial ELISA kits (BioVendor Laboratory Medicine, Inc., Brno, Czech Republic). The precision was 10% (coefficient of variation) for CC-16 and 5% for SP-D, as calculated from duplicate samples. The limit of detection (DL) for CC-16 was 2 µg/L (7% of samples below DL, substituted with DL/2) and for SP-D, it was about 1 μg/L (all samples above DL).

High sensitivity CRP in serum was determined at a clinical commercial laboratory (Fürst Laboratory, Oslo, Norway) using CardioPhase TM, Advia 2400 (Siemens Healthcare Diagnostics Inc., Tarrytown, NY, USA) with a method’s DL of 0.1 mg/L.

### 2.4. Nicotine and Cotinine in Serum

Sample preparation and measurement of nicotine (S-Nico) and cotinine (S-Cot) in serum have been previously described in detail [25]. The method was slightly modified in this study as a different internal standard (cotinine-(methyl-d3)) was used to improve the within- and between assay precisions of the analytical method. The obtained DLs were 1.7 and 7.3 ng/mL for cotinine and nicotine, respectively. The DL was defined as 3 × standard deviation of the blanks. 

### 2.5. Air Sampling and Measurements

The method of air sampling among the drill floor workers has been previously described in detail [10]. In short, air samples were collected by personal sampling in the breathing zone of the exposed subjects and outside personal protective respirators, if used. Oil mist was collected on glass fiber filters mounted in 37-mm closed-face cassettes (CFC) and oil vapor was obtained by charcoal adsorbent tubes connected in series with the CFC. Non-volatile mud components (NVM) were collected on polyvinyl chloride filters, while elemental carbon (EC) and organic carbon (OC) were collected on pre-heated quartz filters mounted in 37-mm Millipore cassettes. The NVM and OC/EC sampling cassettes were fitted with thoracic cyclones. Air flow rates of the pumps were measured with a calibrated rotameter before and after each sampling period. The content of NVM in air and liquid mud samples was used to calculate the concentrations of airborne mud (MUD_Fe_). The analytical procedures have been previously described in detail [10]. No air samples were collected among referents. 

### 2.6. Statistics

Continuous variables were log_10_-transformed to achieve a normal distribution when the skewness of the distributions exceeded 2.0. Geometric means (GM) are presented for these variables. Arithmetic means (AM) or medians are presented otherwise. Student’s *t*-test was used to assess group differences of independent samples. Univariate associations were assessed using least square regression analysis and Pearson’s correlation coefficient calculated as the measure of association. Multiple linear regression analysis (backwards procedure) was used to assess biomarker concentrations at baseline, including the following independent variables simultaneously in one model: exposure category (0/1), age, infection (0/1), body mass index (BMI), time of sampling and current tobacco smoking (0/1). Cross-shift changes in serum CRP and pneumoprotein concentrations were assessed by linear mixed models, including both fixed and random effects (lme function in R, nlme package, version 3.1-122, R Core Team, R Foundation for Statistical Computing, Vienna, Austria). The inclusion of a random intercept for a worker allowed us to take the dependency of repeated observations into account. In addition to the group variable (referent or exposed), the fixed effects included age, BMI, infection, time of sampling and the concentrations of S-Nico and S-Cot. All mixed model analyses were done in R, version 3.2.2. The statistical package SPSS (version 21, IBM Corporation, Somers, NY, USA) was used for all other statistical analyses. Two-tailed *p*-values < 0.05 were considered to be statistically significant. 

## 3. Results

The drill floor workers were younger than the referents, while BMI was similar (Table 1). There were more current smokers and users of snuff among the drill floor workers. Median air concentrations of oil mist and MUD_Fe_ were 0.18 and 0.14 mg/m^3^, respectively.

The drill floor workers had a statistically significantly lower concentration of CRP than the referents at baseline (Table 2). However, when adjusting for differences in age, BMI and prevalence of current smoking, the difference was no longer statistically significant (*p* = 0.19). The concentrations of S-Nico and S-Cot among current snuffers and current smokers were similar in the two studied groups. 

A linear mixed model was applied, which also considered the 14 drill floor workers and 9 referents that were lost to follow-up (Table 3). A significant decline in the serum CC-16 concentrations across the 14-day work period was observed in both groups, while a decline was observed for CRP among the drill floor workers and for SP-D among the referents. However, after adjusting for sampling time, only the reduction in CRP was statistically significant across the 14-day work period among the drill floor workers. There was no statistically significant difference between the drill floor workers and the referents with respect to the alterations of SP-D, CC-16 and CRP across the 14-day work period. No associations were found between various exposure variables and biomarker concentrations among the drill floor workers.

**Figure 1:**
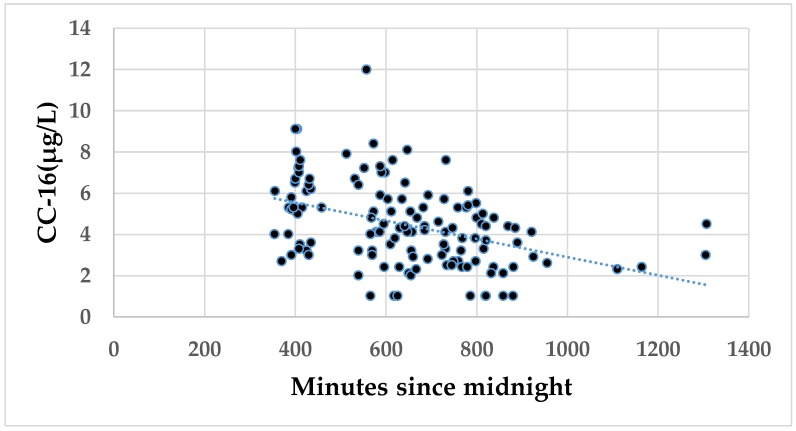
The association between the serum concentrations of club cell protein 16 (CC-16) at baseline and time of blood sampling in minutes after midnight among all participants. The following regression equation was calculated: CC-16 = 7.3 (6.2 to 8.5) −0.004 (−0.006 to −0.003) minutes since midnight. The 95% confidence intervals are shown in brackets. Pearson’s *r* = −0.41; *p* < 0.001.

**Figure 2:**
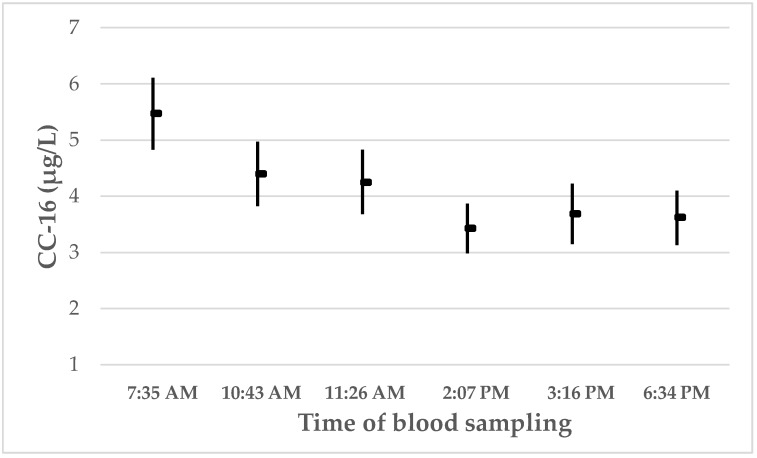
The arithmetic mean (and 95% CI) concentrations of CC-16 in serum according to time of blood sampling. The mean (and min/max) sampling times were: 7:35 AM ^†^ (5:55 AM/9:39 AM); 10:43 AM ^†^ (9:45 AM/0:09 PM); 11:26 AM ^¶^ (10:03 AM/2:03 PM); 2:07 PM ^†^ (0:11 PM/9:47 PM); 3:16 PM ^¶^ (2:10 PM/5:02 PM); and 6:34 PM ^¶^ (5:06 PM/10:19 PM). ^†^ Baseline. ^¶^ Follow-up.

In order to assess the impact of exposure status, sampling time and effect of current smoking on the biomarkers measured at baseline, multiple linear regression was applied (Table 4). The serum concentrations of CC-16 were negatively associated with time of sampling and current smoking. The association between CC-16 concentrations and time of sampling is presented in Figure 1. However, no significant association between time of sampling and serum CC-16 was observed at follow-up. Figure 2 shows the CC-16 concentrations at baseline and at follow-up, respectively, according to time of sampling. The CC-16 concentrations are stratified into three equally large groups both at baseline and at follow-up. It appears that the CC-16 concentrations decline from 7:35 AM until between 11:26 AM and 2:07 PM. Age, BMI and a self-reported current infection were associated with the CRP concentrations, while none of the independent variables was associated with SP-D. 

**Figure 3:**
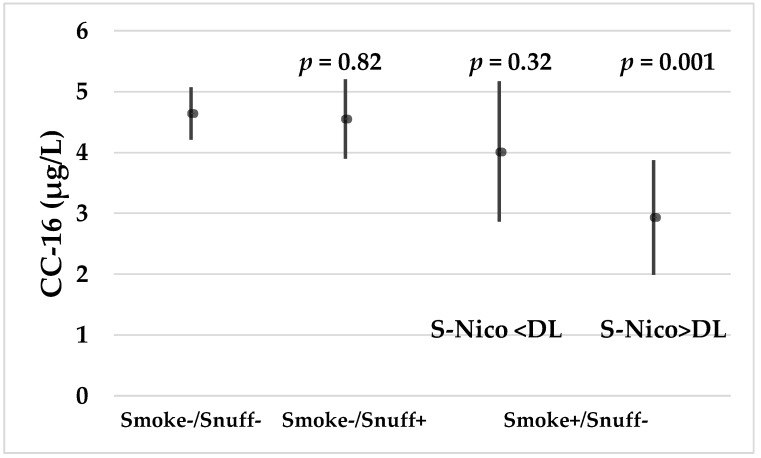
The mean (and 95% CI) concentrations of CC-16 in non-smokers/non-snuffers, non-smokers/snuffers and smokers/non-snuffers according to serum nicotine (S-Nico) concentrations that were higher or lower than the methods’ detection limit. The estimates are adjusted for time of sampling. The *p*-values refer to differences between the group of non-smokers/non-snuffers and the three other groups, respectively.

The concentrations of CC-16 at baseline were not statistically significantly associated with S-Nico and S-Cot, respectively. However, when stratifying the participants according to current smoking and snuffing habits, it appears that those smokers with S-Nico > DL who were not snuffers had significantly lower CC-16 concentrations than both non-smokers/non-snuffers and snuffers. Smokers with S-Nico < DL who were not snuffers had serum CC-16 concentrations comparable to the concentrations of the non-smokers/non-snuffers and snuffers (Figure 3). The GM (and 95% CI) S-Cot concentrations were statistically significantly (*p* < 0.001) lower among smokers with S-Nico < DL (21; 11–40) than among smokers with S-Nico > DL (269; 163–444).

The serum concentrations of SP-D and CC-16 were not associated with measures of pulmonary function at baseline or at follow up. 

## 4. Discussion

This study shows a decline in CC-16 concentrations of drill floor workers and referents across the 14-day work period. However, the decline was found to be statistically insignificant after correcting for the time of sampling and the concentrations of S-Nico and S-Cot. Thus, no alterations in the serum concentrations of the pneumoproteins CC-16 and SP-D were observed that could be related to occupational exposure experienced during oil drilling. Further, the mean concentration of the biomarker of systemic inflammation CRP decreased during the 14-day period of exposure, which is contrary to the hypothesis that CRP could increase due to exposure to mud components. The concentrations of CC-16 were highly dependent on the time of sampling. Although serum concentrations of CC-16 are well known to decrease in current smokers, self-reported smokers with S-Nico < DL had concentrations comparable to that of non-smokers.

The median oil mist and MUD_Fe_ air concentration that the drill floor workers were exposed to were 0.18 mg/m^3^ and 0.14 mg/m^3^, respectively. Some mud components are known irritants to skin and eyes while many drilling muds are alkaline solutions [9,26]. Thus, air exposure to these components have the potential to induce pulmonary effects. However, the air concentrations were not high, which may explain why no alterations in the pneumoprotein concentrations and no increase in CRP concentrations were observed. Elemental carbon, which is a marker of diesel exhaust particulate matter, was present in a limited number of samples at low air concentrations only.

In a previous study, we have shown a decrease in pulmonary function of drill floor workers across the 14-day work period but this decrease was most likely not related to oil mist exposure [11]. Thus, it is of interest that no alterations in the pneumoprotein concentrations could be observed in the same drill floor workers. We are not aware of any previous studies of drill floor workers where serum pneumoprotein concentrations have been assessed. There are also few animal studies addressing this issue. In one study of rats exposed to 2.11 mg/m^3^ of oil mist, a substantial reduction of the SP-D concentration in the bronchioalveolar lavage fluid was observed, while CC-16 was not measured [14]. 

It is generally recognized that exposure to particles may result in systemic inflammation, although a recent review suggested that the evidence is not conclusive [22]. CRP is a biomarker of systemic inflammation. Although the drill floor workers were exposed to particulate matter for 14 days, no increase in CRP was observed. In fact, a slight decrease was measured. One reason may be that this exposure to oil mist (median concentration 0.18 mg/m^3^) combined with a median exposure of 0.14 mg/m^3^ of MUDFe was at levels that were too low and were unable to induce systemic inflammation. 

Although the exposed subjects served as their own referents, it is possible that there could have been differences in other aspects that may have contributed to the results, such as their different diets onshore and offshore. However, to our knowledge, such short-term differences in diet are not known to alter the biomarkers applied in this study.

Due to safety and logistics, a new crew has to be transported off-shore before the old crew can leave the rig for the helicopter transport on-shore after their 14-day work period. Thus, the examinations at follow-up were carried out later in the day than the examinations carried out at baseline. It was therefore important to assess potential confounding related to differences in examination times. A strong association between time of sampling and concentration of CC-16 was observed at baseline, but not at follow-up. This may be due to the later time of sampling at follow-up compared to baseline. Our data indicate that there is a circadian variation in the CC-16 concentrations, with a substantial decline from 7:35 AM to sometime between 11:26 AM and 2:07 PM. The average decline in the CC-16 concentrations is substantial as it changes from nearly 5.5 µg/L at 7:35 AM to around 3.4 µg/L as the lowest measured mean concentration. Thereafter, the concentrations appear to stabilize at least until around 6:30 PM. 

It has been shown previously that serum concentrations of CC-16 are variable during the day. In a small experimental study of 18 healthy subjects who were mainly women, serum CC-16 levels were measured at six different times of the day between 7 AM and 9 PM [27]. That study showed a substantial decline of around 2.5 µg/L from 7 AM to around 2 PM and an increase of around 0.6 µg/L between around 6 PM and 10 PM. The measured concentrations between 11 AM and 6 PM in that study were similar. These data are comparable to the data from our study, both with respect to the magnitude of the decline in the CC-16 concentrations and also in the pattern of the serum concentrations. Helleday et al. [27] did not have any data from around 10:00 PM to 7:00 AM the next morning, but it is a reasonable assumption that the CC-16 concentrations may increase quite substantially during the night. Other studies have also indicated the presence of diurnal variations in the serum CC-16 concentrations [28]. In a study of professional ski waxers, the mean serum CC-16 concentration decreased from 6.7 µg/L at 9:26 AM to 5.9 µg/L at 4:03 PM when they were unexposed [29]. The mechanisms for the circadian variation in the CC-16 concentrations still need to be elucidated.

The difference in serum CC-16 concentrations between the highest and lowest concentrations during the day is substantial. It is therefore important that studies of serum CC-16 concentrations are carefully designed to take these variations into account. Studies where blood is collected before noon may be particularly vulnerable to the variation during the day, because the steepest decline in the concentrations appears to occur in the morning. 

It has been demonstrated that current smoking has a substantial impact on serum concentrations of CC-16 [30,31,32,33,34]. The concentrations of CC-16 are also lower in BAL fluid of smokers as compared to non-smokers [35,36,37]. A significant reduction of club cells in the terminal and respiratory bronchioles was observed in Wistar rats exposed to cigarette smoke [38]. There are indications that epigenetic silencing of CC-16 expression may contribute to the reduced plasma and BAL levels of CC-16 that were observed in smokers [39]. Whether these alterations are reversible upon smoking cessation remains to be elucidated but in this study, former smokers had serum CC-16 concentrations similar to that of never-smokers, which also has been shown previously [40].

The present study shows lower concentrations of serum CC-16 among smokers as compared to non-smokers. However, when stratifying self-reported current smokers according to their S-Nico levels, those with S-Nico < DL have serum CC-16 concentrations similar to those of the non-smokers/non-snuffers. In contrast, smokers with S-Nico > DL have statistically significantly lower CC-16 concentrations as compared to the non-smokers, which is expected. The serum concentration of nicotine has a half-life of approximately 2–3 h [41]. This could imply that the last cigarette was consumed some hours before blood sampling. It is possible that the serum concentrations of CC-16 returned to non-smoker levels within this short time-frame, although this has not been studied. However, a CC-16 plasma half-life of only 16 min was calculated in patients with acute respiratory failure [42]. An alternative explanation could be that current smokers with S-Nico < DL smoke less than smokers with S-Nico > DL. The lower S-Cot concentrations among smokers with S-Nico < DL compared to the smokers with S-Nico > DL could suggest that this could be the case or at least that they were smoking less in the last few days before blood sampling. Serum cotinine has a biological half-life of around 15–20 h [41]. 

Higher concentrations of SP-D in serum have been shown in previous studies of current smokers compared to non-smokers [32,34,43]. This could not be confirmed in the present study. Furthermore, no associations between SP-D and nicotine or cotinine in serum were observed. 

## 5. Conclusions

In summary, no effect of occupational exposure on the serum concentrations of SP-D, CC-16 and CRP was observed. As expected, the concentrations of CC-16 were lower in smokers, but only in those who had detectable S-Nico concentrations. A substantial effect of the time of blood sampling was observed for CC-16, which is important to consider when designing studies assessing CC-16. Conclusions on exposure effects should be based on multiple studies to account for random variation. As this study probably is the only one published so far on pulmonary and systemic inflammation in drill floor workers, further research is recommended.

## Figures and Tables

**Table 1:** Background characteristics of 65 drill floor workers and 65 referents at baseline.

Characteristics	Drill Floor Workers AM ^†^ (Min–Max)	Referents AM (Min–Max)
Age (years) ^a^	30 (19–59)	46 (30–69)
Height (cm)	180.7 (169–196)	181.5 (169–196)
Weight (kg)	85.0 (60–126)	87.4 (67–123)
BMI (kg/m^2^)	26.1 (20.7–39.3)	26.6 (20.2–36.8)
Years of work offshore ^a^	5.8 (0.5–32)	14 (0–35)
Current smokers (%)	31	23
Current snuff users (%)	39	15
Oil mist (mg/m^3^) ^‡,#^	0.18 (<DL–6.0)	-
Oil vapour (mg/m^3^) ^‡,#^	14 (<DL–120)	-
MUD_Fe_ (mg/m^3^) ^‡,^^☼^	0.14 (<DL–2.4)	-

^†^ arithmetic mean; ^‡^ median; ^a^ *p* < 0.05; ^#^ based on 61 air samples; ^☼^ based on 58 air samples.

**Table 2:** The serum concentrations of C-reactive protein (CRP), club cell protein 16 (CC-16), surfactant protein D (SP-D) and nicotine and cotinine at baseline in 65 drill floor workers and 65 referents. Mean concentrations adjusted for age, current smoking habits and BMI are shown in brackets.

Biomarkers	Drill Floor Workers		Referents		
	AM ^†^	Min–Max	AM	Min–Max	*p*
CRP ^‡^ (mg/L)	0.9 (1.0)	0.1–12	1.4 (1.3)	0.2–16	0.02
CC-16 (µg/L)	4.5 (4.6)	1.0–9.1	4.4 (4.3)	1.0–12	0.62
SPD ^‡^ (µg/L)	166 (167)	70–886	176 (174)	59–448	0.45
Current smokers (*n*)	20	-	15	-	-
S-nicotine (µg/L)	14.0	<DL–53	20.4	<DL–54	0.18
S-cotinine (µg/L)	205	<DL–382	236	4.3–438	0.50
Current snuff-users (*n*)	24	-	9	-	-
S-nicotine (µg/L)	14.8	<DL–54	19.2	<DL–39	0.40
S-cotinine (µg/L)	315	<DL–1275	340	123–500	0.79

^†^ arithmetic mean; ^‡^ geometric mean.

**Table 3:** Changes in the serum concentrations of C-reactive protein (CRP), surfactant protein D (SP-D) and club cell protein 16 (CC-16) after being adjusted for age, BMI, infection, nicotine and cotinine across a 14-day work period of drill floor workers and referents. Data are also presented for the additional adjustment for time of blood sampling.

Biomarkers		Drill Floor Workers (*n* = 65/51) ^a^				Referents (*n* = 64/55) ^a,b^				Drill Floor Workers vs. Referents			
	Bio-Marker	Esti-Mate	Lower	Upper	*p*	Esti-Mate	Lower	Upper	*p*	Esti-Mate	Lower	Upper	*p*
Not adjusted for time of day	CRP ^c^	–0.12	−0.23	0.00	0.045	−0.07	−0.18	0.05	0.25	−0.05	−0.21	0.11	0.53
	SPD ^c^	−0.02	−0.05	0.01	0.12	−0.03	−0.06	−0.01	0.017	0.01	−0.03	0.05	0.56
	CC16	−0.71	−1.06	−0.36	0.0001	−0.59	−0.93	−0.24	0.001	−0.12	−0.62	0.37	0.62
Adjusted for time of day	CRP ^c^	−0.14	−0.27	−0.01	0.038	−0.09	−0.23	0.05	0.19	−0.04	−0.21	0.12	0.58
	SPD ^c^	−0.01	−0.05	0.02	0.36	−0.02	−0.06	0.01	0.2	0.01	−0.03	0.05	0.64
	CC16	−0.35	−0.77	0.07	0.1	−0.12	−0.57	0.33	0.61	−0.23	−0.73	0.26	0.36

^a^ Number of observations before and after shift (with complete covariate information). ^b^ CRP had *n* = 64/54 and SP-D had *n* = 63/54. ^c^ Log_10_-transformed.

**Table 4:** Statistically significant results from multiple linear regression analysis (backward procedure). Dependent variables were the concentrations of C-reactive protein (CRP), club cell protein 16 (CC-16) and surfactant protein D (SP-D) at baseline. Independent variables were exposure category (Expo) (0/1), age (years), infection (0/1), body mass index (BMI) (kg/m^2^), sampling time (minutes since midnight) and current tobacco smoking (0/1). Beta-coefficients and corresponding *p*-values are presented.

Biomarkers	Expo	Age	BMI	Smoking	Sampling Time	Infection	Mult. *r*
CRP (mg/L) ^†^	-	0.008 *	0.04 ***	-	-	0.28 **	0.45 ***
SP-D (µg/L) ^†^	-	-	-	-	-	-	No model
CC-16 (µg/L)	-	-	-	−0.73 *	−0.004 ***	-	0.45 ***

^†^ variable transformed to lg; *** *p* < 0.001; ** *p* < 0.01; * *p* < 0.05.
